# A new gene inventory of the ubiquitin and ubiquitin-like conjugation pathways in *Giardia intestinalis*

**DOI:** 10.1590/0074-02760190242

**Published:** 2020-02-21

**Authors:** Isabel Cristina Castellanos, Eliana Patricia Calvo, Moisés Wasserman

**Affiliations:** 1 Universidad Escuela de Administración de Negocios, Departamento de Ciencias Básicas, Bogotá, Colombia; 2 Universidad El Bosque, Laboratorio de Virología, Bogotá, Colombia; 3 Universidad Nacional de Colombia, Laboratorio de Investigaciones Básicas en Bioquímica, Bogotá, Colombia

**Keywords:** ubiquitin, ubiquitin-like protein, ubiquitin ligase, deubiquitinating enzymes, Giardia, hidden Markov models

## Abstract

**BACKGROUND:**

Ubiquitin (Ub) and Ub-*like* proteins (Ub-L) are critical regulators of complex cellular processes such as the cell cycle, DNA repair, transcription, chromatin remodeling, signal translation, and protein degradation. *Giardia intestinalis* possesses an experimentally proven Ub-conjugation system; however, a limited number of enzymes involved in this process were identified using basic local alignment search tool (BLAST). This is due to the limitations of BLAST’s ability to identify homologous functional regions when similarity between the sequences dips to < 30%. In addition Ub-Ls and their conjugating enzymes have not been fully elucidated in *Giardia*.

**OBJETIVE:**

To identify the enzymes involved in the Ub and Ub-Ls conjugation processes using intelligent systems based on the hidden Markov models (HMMs).

**METHODS:**

We performed an HMM search of functional Pfam domains found in the key enzymes of these pathways in *Giardia’s* proteome. Each open reading frame identified was analysed by sequence homology, domain architecture, and transcription levels.

**FINDINGS:**

We identified 118 genes, 106 of which corresponded to the ubiquitination process (Ub, E1, E2, E3, and DUB enzymes). The E3 ligase group was the largest group with 82 members; 71 of which harbored a characteristic RING domain. Four Ub-Ls were identified and the conjugation enzymes for NEDD8 and URM1 were described for first time. The 3D model for Ub-Ls displayed the β-grasp fold typical. Furthermore, our sequence analysis for the corresponding activating enzymes detected the essential motifs required for conjugation.

**MAIN CONCLUSIONS:**

Our findings highlight the complexity of *Giardia*’s Ub-conjugation system, which is drastically different from that previously reported, and provides evidence for the presence of NEDDylation and URMylation enzymes in the genome and transcriptome of *G. intestinalis*.

Ubiquitin (Ub) and ubiquitin-like modifiers (Ub-Ls) are small proteins that covalently attach to protein substrates and regulate various cellular processes such as the cell cycle, endocytosis, signaling pathways, intracellular trafficking, DNA repair and transcription, among others.^1^

Ub and Ub-Ls share two common features: a β-grasp fold composed of a five-stranded β-sheet and a C-terminal diglycine motif (GG) used for conjugation to target proteins. Currently, the Ub-L family includes 10 members: small ubiquitin modifier (SUMO), neural precursor cell expressed developmentally downregulated 8 (NEDD8) or Related to Ubiquitin 1 (RUB 1) in yeast, Ubiquitin-Related Modifier-1 (URM1), Ubiquitin-fold Modifier 1 (UFM1), autophagy-related proteins 8 and 12 (ATG8 and ATG12), interferon-stimulated gene 15 (ISG15), human leukocyte antigen (HLA)-F adjacent transcript 10 (FAT10), fan ubiquitin-like protein 1 (FUB1), and histone mono-ubiquitination 1 (HUB1).^2^

The first step in the Ub-conjugation cascade is activation, which is mediated by the E1 protein (UBA1 in the budding yeast). Further, Ub is transferred to a Ub-conjugating enzyme or E2 (UBC) through a transesterification reaction. Finally, Ub ligase or E3 directly or indirectly transfers Ub to the substrate.^3^

E3 enzymes are a wide and diverse group of proteins that can be classified into three groups according to conserved structural domains and the transfer mechanism of Ub to the substrate. The family of Homologous to E6-associated protein carboxyl terminus (HECT) uses an indirect or two-step mechanism in which Ub is transferred from E2 to E3 and then to the substrate. The family of Really Interesting New Gene (RING) and RING-related E3s have a domain containing short motifs of cysteines and histidines, which coordinate two zinc ions (Zf-C3HC4, Zf-UBR, Zf-B Box, PHD, and Zf-Mynd domains) and act as scaffolds for conjugation, thus promoting direct transfer of Ub from E2 to the substrate.^3^

Ubiquitination is a reversible process in which the deubiquitinating enzymes (DUBs) hydrolyze poly-Ub chains or remove Ub molecules. The human genome codifies approximately 90 DUBs that are classified into six families: ubiquitin C-terminal hydrolases (UCHs), ubiquitin-specific proteases (USPs), Machado-Joseph Disease (MJD), Permuted Papain fold Peptidases of DsRNA viruses and Eukaryotes (PPPDE), ovarian tumor (OTU), metalloproteases with a JAMM/MPN motif, and the recently described motif interacting with Ub-containing protein (MINDY-4).^4^

*Giardia intestinalis* is a protozoan parasite that is considered to be an early divergent eukaryote; it lacks typical eukaryotic organelles such as mitochondria, peroxisomes, and Golgi apparatus. *Giardia* is an important eukaryotic model because it could have only the key components of the principal regulation systems that characterize higher eukaryotes.^5^ Our laboratory previously reported that a large number of ubiquitinated proteins exist during the motile, active metabolic, and replicative stage of *Giardia* (trophozoite); 151 proteins distributed over 14 functional categories were identified. However, in the infective stage (cyst), only 55 ubiquitinated substrates were observed. Despite this marked decrease, ubiquitination of enzymes involved in cyst wall biogenesis suggested that Ub modification plays a crucial role in this stage of the cell cycle.^6^ Therefore, *Giardia* might be a suitable biological model to define the fundamental elements of the Ub-conjugation pathway. The proteasome components have recently been analysed using bioinformatics, confirming findings reported earlier where a remarkable conservation was observed.^7^

Previous studies have identified three genes for Ub, one E1 enzyme, 11 E2 enzymes, four E3 ligases, and 9 DUBs;^8^ ^,^ ^9^ ^,^ ^10^ ^,^ ^11^ ^,^ ^12^ ^,^ ^13^ however, the most divergent genes may have been overlooked. Herein, we performed an exhaustive search using an intelligent systems approach based on hidden Markov models (HMMs) with profiles from the Pfam and Superfamily databases. Approximately 120 genes were identified, 88 of which correspond to new findings; among these genes, 76 were E3 ligases. Furthermore, we identified NEDD8 and URM1 conjugation pathways.

## MATERIALS AND METHODS

*Inventory building* - The full proteome database from *G. intestinalis* was downloaded from Eupath database version 5.0 (available at http://giardiadb.org). In addition, 66 Pfam HMM profiles associated with Ub and Ub-Ls conjugation systems were selected and downloaded from the Pfam database version 31.0 (http://pfam.xfam.org/) (Table I). The HMMER package version 3.1 (http://hmmer.org) was used to search each Pfam profile against the entire proteome dataset using the hmmsearch tool and a threshold E-value ≤ 0.1. The repetitive tasks were automated using a perl script.

Each result was analysed for the respective domain, and other structural features were verified by basic local alignment search tool (BLAST) searches in the EMBL Pfam database (http://pfam.xfam.org/) and SMART analysis program (http://smart.embl-heidelberg.de/smart/set_mode.cgi). Finally, each sequence identified in *Giardia* proteome was used as a query on BLASTp tool from www.giardiadb.org using the UniProtKB/Swiss-Prot database to identify orthologs.

**TABLE I:** Pfam hidden Markov models (HMMs) profiles used as queries against *Giardia* proteome database

	Domain	Profile
Ubiquitin Ub _L	Ubiquitin	PF00240;PF14560; PF14836
	Ribosomal L40e	PF01020
	Ribosomal S27a	PF01599
	This	PF02597
	ATG8	PF02990
	ATG12	PF04110
	UFM1	PF03671
	URM1	PF09138
Activating enzymes	ThiF family	PF00899
	Uba 5	PF16190
	Ubiquitin fold domain	PF09358
	Ubiquitin activating	PF16195
	SUMO activating	PF14732
Conjugating enzymes	UQ_con	PF00179
	UFC1	PF08694
	UAE	PF14732; PF08694
HECT ligases	HECT	PF00632; PF11547
RING ligases	zf-C3HC4	PF00097; PF13920; PF13923; PF15227; PF09743; PF15815; PF01485; PF16562; PF09288; PF12483; PF14496; PF16685; PF15926; PF09046; PF15303;
	zf-C2H2	PF00096
	zf-RING_UBOX,	PF13445
	BRE1	PF08647
	Zf-B-box	PF00643
	Zinc finger, ZZ type	PF00569
	Zf- MYND	PF01753
	Zf-UBR	PF02207
	U-box domain	PF04564
	PHD-finger	PF00628;PF13831; PF16866;
	F-box;	PF00646
	APC10	PF03256
	APC13p	PF05839
	APC15p	PF05841
	APC subunit 2	PF08672
	Cullin family	PF00888
	Skp1 family	PF01466; PF09743
	UFM1 ligase	SSF57850 Superfamily DB
	RING/U-box,	
Deubiquitinating (DUB)	UCH	PF01088, PF00443; PF06337
	OTU	PF02338
	PPPDE	PF05903
	Peptidase family C78	PF07910
	MINDY	PF13898

*Expression analysis* - To analyse gene expression profiles, RNA-seq and microarray datasets were employed. The data were downloaded from NCBI’s gene expression omnibus (GEO), with accession numbers GSE36490 and GSE25460 respectively, and parsed using in-house perl scripts.

*Protein model building and evaluation* - 3D models for Ub and Ub-Ls were obtained using Phyre2 (www.sbg.bio.ic.ac.uk/phyre2/). 2LRW, 2QJL, 1YX5, and 1A5R PDB structures were used to model Ub, URM1, NEDD8, and SUMO, respectively. The predicted models were subjected to energy minimisation using YASARA (http://www.yasara.org/), and the stereochemical stability was verified using PROCHECK and ProSA analysis (http://www.ebi.ac.uk/thornton-srv/databases/pdbsum; https://prosa.services.came.sbg.ac.at/prosa.php). Ramachandran plots were computed using Rampage to determine the stereochemical quality and predicted accuracy of the structures.

*Phylogenetic analysis* - Protein sequences were retrieved from Uniprot and aligned using CLUSTAL Omega (https://www.ebi.ac.uk/Tools/msa/clustalo/). A neighbor-joining phylogenetic tree was constructed using the MEGA 7 program. Bootstrap values were obtained from 1000 replicates.

## RESULTS

Our methodology for searching proteins that compose Ub and Ub-L conjugation systems in *G. intestinalis* identified 118 sequences that were classified into five groups: Ub and Ub-*like*, E1 and E1*-like*, E2, E3, and DUB enzymes (Table II).

**Fig. 1:**
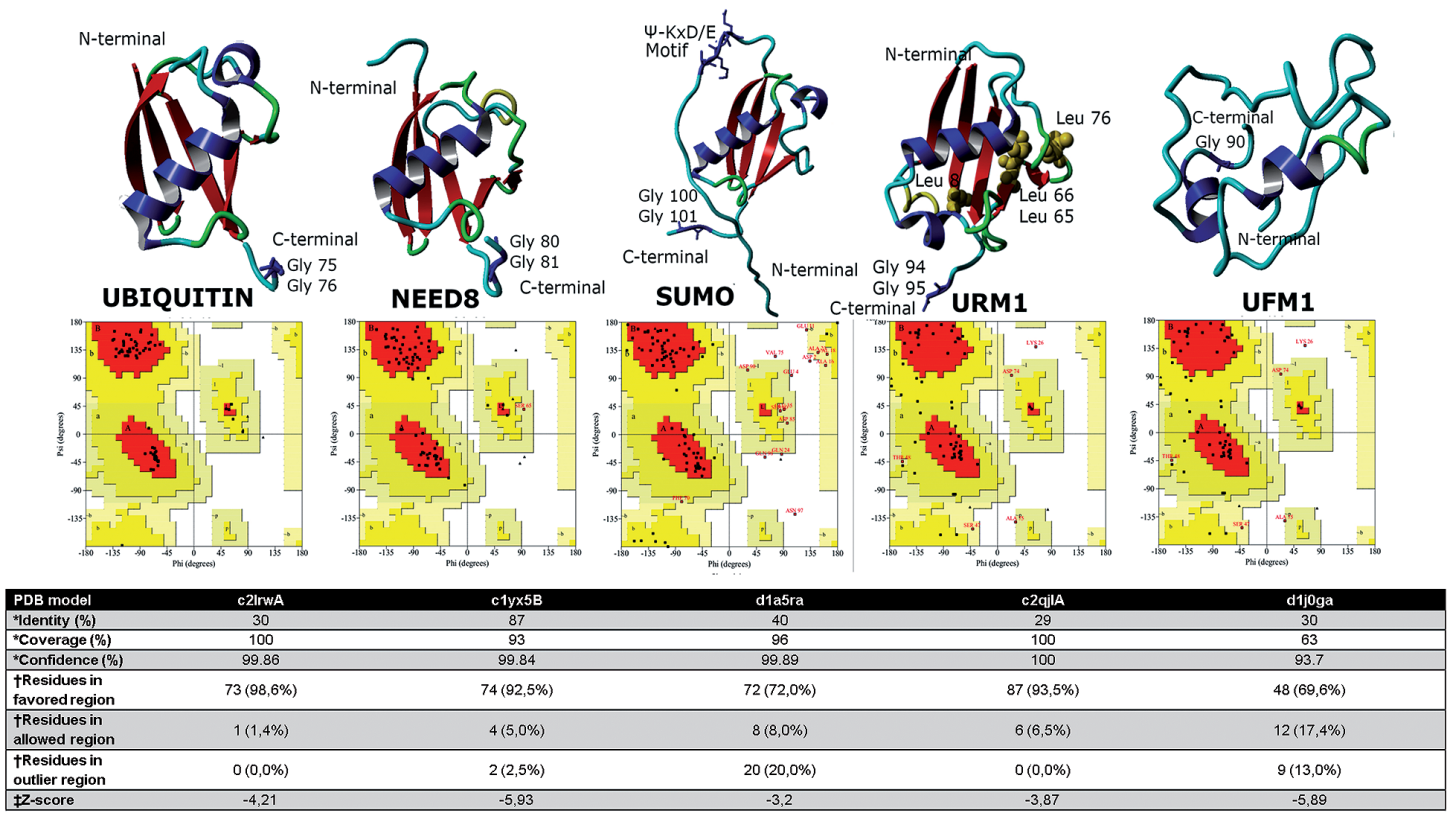
homology modeling and evaluation of Ubiquitin (Ub) and Ub-like proteins (Ub-Ls) structures. Three-dimensional structure of proteins was determined using the homology modeling program Phyre2*, whereas the reliability of the model was assessed via Ramachandran plot analysis^†^. Ribbon diagrams and quality plots are shown. Red color areas indicate the favored regions, yellow color areas indicate the allowed regions, and white colour areas indicate the generously allowed regions. Statistical parameters are listed in the table underneath. Overall quality score was calculated via ProSA^‡^. For each model, the z-score was within the range of scores of all experimentally determined proteins in the current PDB.

The first group contains seven sequences: one for free Ub, two for fused Ub (Ub-L40 and Ub-S27), and four for Ub-*like* proteins: SUMO, URM1, RUB1, which is an ortholog of mammalian NEDD8, and UFM1. Among proteins identified, RUB1 is the closest to Ub (41% identical), whereas UFM1 is the least similar, with 16% identity. To characterise these sequences structurally, three-dimensional structure predictions for each protein were performed. The predicted structures for Ubiquitin, SUMO, NEDD8, and URM1 were similar to Ub-Ls as they possessed a β grasp fold; the characteristic diglycine motif at the C-terminus and the hydrophobic core (Ile-66, Leu-65, and Leu-74) which is conserved in URM1 orthologs, was also identified.^2^ Ramachandran plots were then built to assess the quality of the structures. The plots indicated that the distribution of residues in the allowed and disallowed regions as well as the plot analysis predicting the stability of the models are within reliable ranges (Fig. 1).

**TABLE II:** Proteins identified by profile hidden Markov models (HMMs) search

Group	Class	BLASTP reciprocal best-hit					
		Gen ID	Reference	Protein	Organism	Similarity (%)	Identity (%)
Ubiquitin	Ub	GL50803_8843	8	Ubiquitin	*Homo sapiens*	70	39
	UbL40	GL50803_5665	9	Ubiquitin-60S ribosomal protein L40	*Trypanosoma brucei*	69	48
	UbS27	GL50803_16298	10	Ubiquitin-40S ribosomal protein S27a	*Kluyveromyces lactis*	72	57
Ubiquitin *Like*	SUMO	GL50803_7760	13	Small ubiquitin-related modifier 1 (SUMO)	*H. sapiens*	64	47
	NEDD8	GL50803_7110		Related to Ubiquitin 1(RUB 1) or NEDD8	*Arabidopsis thaliana*	95	84
	URM1	GL50803_11884	11	Ubiquitin-related modifier 1 (URM1)	*Schizosaccharomyces pombe*	55	35
	UFM1	GL50803_104982		Ubiquitin-fold modifier 1 (UFM1)	*Pongo abelii*	51	29
E1	UBA1	GL50803_10661	10	Ubiquitin-activating enzyme E1	*S. pombe*	52	35
E1-*Like*	UBA2	GL50803_6288	13	SUMO-activating enzyme subunit 2	*Dictyostelium discoideum*	46	23
	UBA3	GL50803_4083		NEDD8-activating enzyme E1 catalytic subunit	*S. pombe*	49	31
	UBA4	GL50803_12853		Adenylyltransferase and sulfurtransferase UBA4	*S. pombe*	55	39
	MoeB	GL50803_11436		Molybdopterin biosynthesis MoeB protein	*Trepomonas sp. PC1*	67	49
E2		GL50803_15252	10	Ubiquitin-conjugating enzyme E2 5A	*Oryza sativa*	76	64
		GL50803_12950	10	Ubiquitin-conjugating enzyme E2 2	*Ashbya gossypii*	63	45
		GL50803_15162	10	Ubiquitin-conjugating enzyme E2 2	*Triticum aestivum*	66	48
		GL50803_3978	9	Ubiquitin-conjugating enzyme E2 PEX 4	*A. thaliana*	60	41
		GL50803_27055	10	Ubiquitin-conjugating enzyme E2 S	*Drosophila simulans*	61	30
		GL50803_6524	9	Ubiquitin-conjugating enzyme E2 J2	*H. sapiens*	59	41
		GL50803_3171	9	Ubiquitin-conjugating enzyme E2 UBC14	*A. thaliana*	73	54
		GL50803_2876	10	Ubiquitin-conjugating enzyme E2 UBC2	*T. aestivum*	69	48
		GL50803_5921	10	Ubiquitin-conjugating enzyme E2 J2	*H. sapiens*	61	39
		GL50803_31576	9	NEDD8-conjugating enzyme UBC12	*Yarrowia lipolytica*	58	33
		GL50803_24068	9	SUMO-conjugating enzymeUBC9	*S. pombe*	68	55
		GL50803_8638		ubiquitin-conjugating enzyme UBC6	*S. pombe*	64	43
E3	HECT	GL50803_137754	10	E3 Ubiquitin protein ligase E3A	*H. sapiens*	54	36
		GL50803_17386	10	E6AP HECT Catalitic domain E3 Ligase	*H. sapiens*	53	34
		GL50803_16321		E3 ubiquitin protein ligase Pub1	*S. pombe*	45	28
		GL50803_32730		HECT-type E3 ubiquitin transferase	*Caenorhabditis elegans*	44	31
		GL50803_3117		HECT type ubiquitin ligase	*Eimeria maxima*	47	31
	RING finger domain: Zf-C3HC4	GL50803_16541		E3 ligase XBAT32; Ankyrin repeat domain and RING finger	*A. thaliana*	46	29
		GL50803_4843		E3 ligase XBAT33	*A. thaliana*	44	30
		GL50803_16227		E3 ligase XBAT33	*A. thaliana*	44	33
		GL50803_14203		E3 ligase-XBOS33	*O. sativa Japonica*	41	30
		GL50803_17552		E3 ligase XBAT34	*A. thaliana*	49	32
		GL50803_4320		E3 ligase XBOS36	*O. sativa Japonica*	38	26
		GL50803_10605		E3 ligase XBOS31	*O. sativa Japonica*	46	34
		GL50803_14934		Ser/Thr-protein kinase NEK7;	*H. sapiens*	49	32
		GL50803_2351		E3 ubiquitin-protein ligase mind-bomb	*D. melanogaster*	53	37
		GL50803_6284		E3 ubiquitin-protein ligase mind-bomb	*D. melanogaster*	46	31
		GL50803_21799		E3 ubiquitin-protein ligase mind-bomb	*D. melanogaster*	41	25
		GL50803_33807		E3 ubiquitin-protein ligase mind-bomb 1 (MIB1)	*Danio rerio*	47	31
		GL50803_6602		E3 ubiquitin-protein ligase MIB1	*H. sapiens*	45	28
		GL50803_4329		E3 ubiquitin-protein ligase MIB1	*Mus musculus*	43	23
		GL50803_10703		E3 ubiquitin-protein ligase MIB1	*Xenopus laevis*	44	28
		GL50803_8438		E3 ubiquitin-protein ligase MIB1	*D. rerio*	41	26
		GL50803_89845		E3 ubiquitin-protein ligase MIB1	*X. laevis*	41	25
		GL50803_115054		E3 ubiquitin-protein ligase MIB 1	*X. laevis*	40	24
		GL50803_15412		E3 ubiquitin-protein ligase MIB1	*M. musculus*	40	22
		GL50803_13901		E3 ubiquitin-protein ligase Mind bomb 2 (MIB2)	*H. sapiens*	48	33
		GL50803_6650		E3 ubiquitin-protein ligase MIB2	*Gallus gallus*	42	29
		GL50803_11389		E3 ubiquitin-protein ligase MIB2	*G. gallus*	39	24
		GL50803_8325		Baculoviral IAP Repeat-containing Protein 7; E3 Ligase BIRC3	*H. sapiens*	71	50
		GL50803_14206		Baculoviral IAP repeat-containing protein 2	*H. sapiens*	61	45
		GL50803_7021		Baculoviral IAP repeat-containing protein 2	*M. musculus*	50	34
		GL50803_94662		Baculoviral IAP repeat-containing protein 2	*M. musculus*	46	28
		GL50803_6589		Baculoviral IAP repeat-containing protein 1	*A. californica nucleopolyhedrovirus*	58	43
		GL50803_113284		E3 ubiquitin-protein ligase LUL4	*A. thaliana*	60	39
		GL50803_92983		E3 ubiquitin-protein ligase LUL4	A. thaliana	56	39
		GL50803_114442		E3 ubiquitin-protein ligase LUL4	A. thaliana	56	39
		GL50803_17329		E3 ubiquitin-protein ligase TRIM5	*H. sapiens*	56	34
		GL50803_21233		E3 ubiquitin-protein ligase TRIM38	*B. taurus*	39	23
		GL50803_34160		E3 ubiquitin Tripartite motif-containing protein 43C	*M. musculus*	58	42
		GL50803_4837		E3 ubiquitin Tripartite motif-containing protein 46	*M. musculus*	56	36
		GL50803_95254		E3 ubiquitin-protein ligase TRIM58	*H. sapiens*	47	38
		GL50803_106320		RING finger and CHY zinc finger domain E3 Pirh2	*M. musculus*	47	35
		GL50803_101011		RING finger and CHY zinc finger domain E3 Pirh2	*M. musculus*	41	30
		GL50803_15187		RING finger and CHY zinc finger domain-	*H. sapiens*	52	37
		GL50803_103659		RING finger and CHY zinc finger domain-	*H. sapiens*	48	34
		GL50803_1774		RING finger and CHY zinc finger domain-	*S. pombe*	51	37
		GL50803_11054		E3 ubiquitin-protein ligase RNF 168	*X. laevis*	63	45
		GL50803_3146		E3 ubiquitin-protein ligase RNF 213	*D. rerio*	57	36
		GL50803_3279		E3 ubiquitin-protein ligase RING1-like	*A. thaliana*	59	47
		GL50803_16157		E3 RING finger protein	*Tetrahymena thermophila*	45	27
		GL50803_15868		E3 ubiquitin-protein ligase Zswim2	*M. musculus*	45	28
		GL50803_9850		Postreplication repair E3 ubiquitin-protein ligase Rad18	*S. pombe*	49	35
		GL50803_14796		E3 ubiquitin-protein ligase; PDZ protein 3	*H. sapiens*	52	33
		GL50803_4343		E3 ubiquitin-protein ligase hel2 (histone E3 ligase)	*S. pombe*	46	32
		GL50803_95918		E3 ubiquitin ligase bre1	*Neurospora crassa*	57	36
		GL50803_8731	10	E3 ubiquitin ligase RMA1	*A. thaliana*	52	34
		GL50803_113625		Transcriptional adapter 2-beta	*X. laevis*	46	28
		GL50803_21398		E3 ubiquitin-protein ligase ARI11	*A. thaliana*	63	48
		GL50803_9155		E3 ubiquitin-protein ligase RNF181	*X. laevis*	54	33
		GL50803_13737		Probable E3 ubiquitin-protein ligase LUL4	*A. thaliana*	51	34
		GL50803_11052		Probable E3 ubiquitin-protein ligase LUL4	*A. thaliana*	54	32
		GL50803_21792		E3 ubiquitin-protein ligase SP1	*A. thaliana*	54	37
		GL50803_4430		E3 ubiquitin-protein ligase XIAP	*X. laevis*	51	35
		GL50803_16687		Baculoviral IAP repeat-containing protein 3	*H. sapiens*	62	45
		GL50803_17109		E3 ubiquitin transferase Pep5	*M. musculus*	47	27
		GL50803_11930		SUMO-protein ligase pli1; MIZ/SP-RING	*S. pombe*	54	33
		GL50803_10261		SUMO-protein ligase PIAL2; MIZ/SP-RING	*A. thaliana*	46	25
		GL50803_21622		RING finger protein 151	*Bos taurus*	61	52
		GL50803_9807		E3 ubiquitin-protein ligase PRT1/N-end rule pathway	*A. thaliana*	54	34
		GL50803_14241		E3 ubiquitin-protein ligase PRT1/N-end rule pathway	*A. thaliana*	52	40
		GL50803_4897		RING finger protein 32	*H. sapiens*	44	26
		GL50803_16464		Transmembrane E3 ubiquitin-protein ligase FLY1	*A. thaliana*	54	33
		GL50803_4044		Transmembrane E3 ubiquitin-protein ligase 1 TUL1	*Saccharomyces cerevisiae*	55	47
		GL50803_7356		E3 ubiquitin-protein ligase LRSAM1	*H. sapiens*	42	25
		GL50803_16475		Structure-specific endonuclease subunit SLX1	*D. rerio*	50	36
		GL50803_8432		E3 Ubiquitin ligase APC 11 / Ring Box protein 1 (RBX)	*M. musculus*	44	28
		GL50803_8241		RING-box protein 2 (RBX) / Nedd8 E3 Ligase	*M. musculus*	55	39
	Zf-UBR	GL50803_13708	10	E3 ubiquitin-protein ligase UBR1/E3 alfa	*H. sapiens*	53	39
	Zf-B Box	GL50803_8140		E3 ubiquitin-protein ligase arc-1	*C. elegans*	45	27
	Zf-Mynd	GL50803_17543		Zinc finger MYND domain-containing protein 10	*H. sapiens*	41	24
		GL50803_17492		SET domain and MYND-type zinc finger protein 6	*S. pombe*	60	48
	PHD-finger	GL50803_15559		Jade PHD finger protein 1	*H. sapiens*	48	37
		GL50803_8381		PHD-ARID4 (ARID domain-containing protein 4)	*A. thaliana*	44	33
DUB	UCH	GL50803_14460	10	Ubiquitin carboxyl-terminal hydrolase 4	*S. cerevisiae*	46	32
		GL50803_16090	10	Ubiquitin carboxyl-terminal hydrolase 8	*H. sapiens*	39	26
		GL50803_5533	10	Ubiquitin carboxyl-terminal hydrolase 17-like	*M. musculus*	40	24
		GL50803_6317	9	Ubiquitin carboxyl-terminal hydrolase 44	*D. rerio*	55	37
		GL50803_102710	9	Ubiquitin carboxyl-terminal hydrolase 14	*S. pombe*	42	23
		GL50803_8189	10	Ubiquitin carboxyl-terminal hydrolase 6	*A. thaliana*	41	24
		GL50803_16438	13	Sentrin specific protease SENP5 (SUMOylase 1)	*M. musculus*	52	29
		GL50803_10218		Ubiquitin carboxyl-terminal hydrolase L3 (deNEDDylase)	*Nanorana parkeri*	42	22
	JAMM/ MPN	GL50803_16823	11	Ubiquitin carboxyl-terminal hydrolase RPN11	*S. pombe*	59	39
	PPPDE	GL50803_24425	12	Desumoylating isopeptidase 1	*H. sapiens*	64	40
	OTU	GL50803_88556		Otubain	*H. sapiens*	41	21
	MINDY	GL50803_7349		Ubiquitin carboxyl-terminal hydrolase MINDY-4	*B. taurus*	47	31

**Fig. 2:**
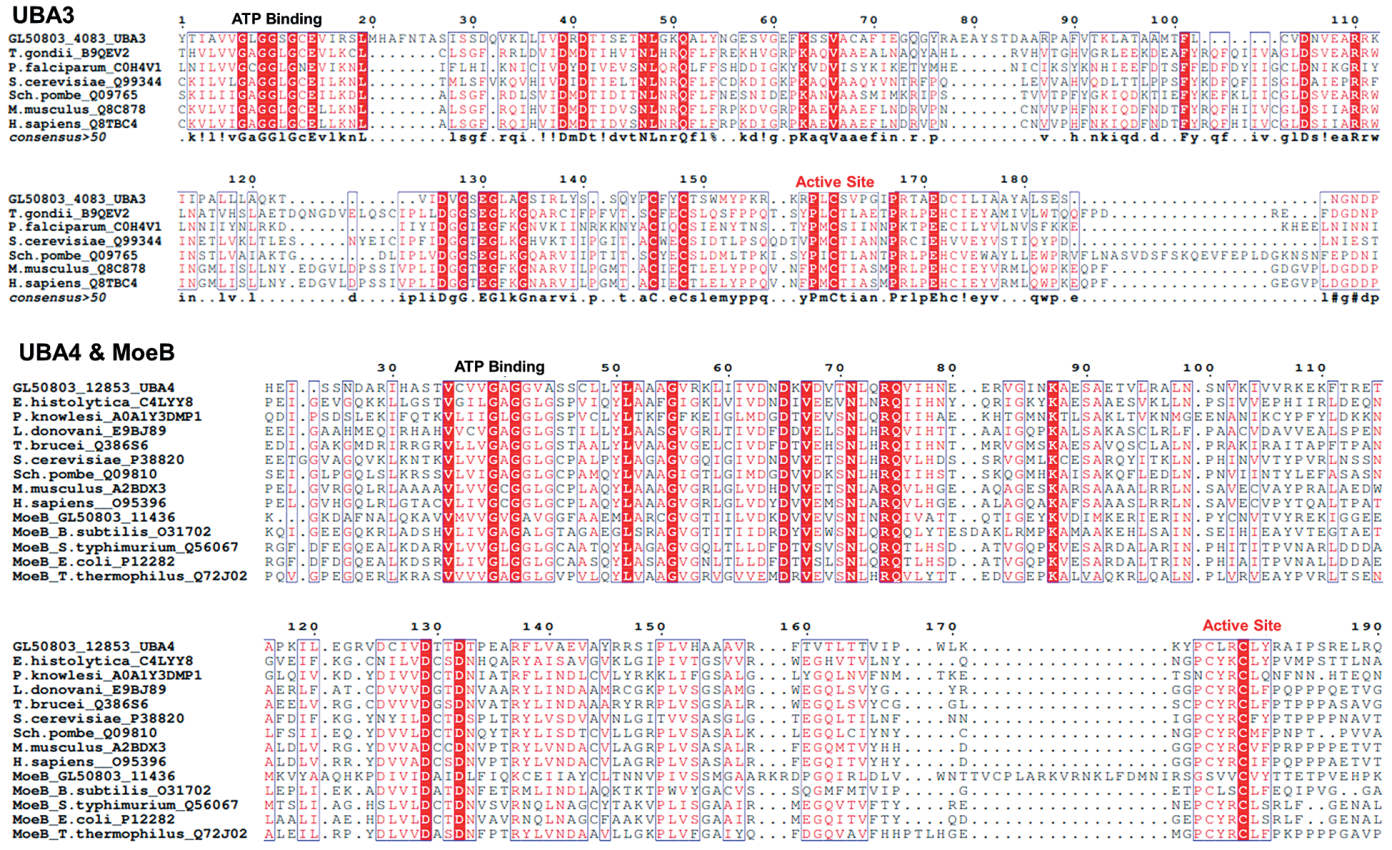
multiple sequence alignment for activating enzymes. Sequence alignment was developed for UBA3 sequences from *Toxolplasma gondii, Plasmodium falciparum*, *Saccharomyces cerevisiae*, *Schizosaccharomyces pombe*, *Mus musculus*, *Homo sapiens*, and GL50803_4083. For UBA 4 and MoeB alignment, sequences from *Entamoeba histolytica*, *Plasmodium Knowlesi*, *Leishmania donovani*, *Trypanosoma brucei*, *S. cerevisiae*, *S. pombe*, *M. musculus*, *H. sapiens*, *Bacilus subtilis*, *Salmonella typhimurium*, *Escherichia coli*, *Thermus thermophiles*, GL50803_12853 and GL50803_11436 were analyzed. The ATP binding site and catalytic cysteine at the activation domain are indicated.

The second group includes proteins identified with the ThiF domain, which is characteristic of Ub activating enzymes and members of the bacterial ThiF/MoeB/HesA family. E1 enzymes for Ub (UBA1), SUMO (UBA2), NEDD8 (UBA3), URM1 (UBA4), and one ortholog of MoeB were identified (Table II). These enzymes harbor two catalytic activities required for activation; adenylation and thioester bond formation. The presence of the nucleotide binding motif, GXGXXGCE, and the catalytic cysteine motif, PZ**C**TXXXXP, which are conserved among canonical E1s (UBA1, 2, and 3) in sequences GL50803_4083, GL50803_10661, and GL50803_6288 confirming our findings (Fig. 2). The sequences GL50803_12853 and GL50803_11436 exhibited some degree of similarity with the noncanonical E1 UBA4 and the prokaryotic protein MoeB, respectively. UBA4 (MOCS3 in human) has dual functions in both protein urmylation and in sulfur transport within the tRNA thiolation pathways. MoeB activates MoaD through its C-terminal end during the first step to incorporate sulfur during molybdenum cofactor biosynthesis.^14^ The comparison of *Giardia* sequences with their orthologs demonstrated that UBA4 is more closely related to MoeB than to canonical E1s (Fig. 2).

E2 enzymes are characterised by a highly conserved domain of approximately 150 amino acids or UBC, which contains a conserved catalytic cysteine and interacts with E1. E2 are classified into four groups based on the existence of additional extensions flanking the UBC domain that confer functional differences. Class I has only the UBC domain, classes II and III have one extension (N- or C-terminal), and class IV has both. In *Giardia*, we found 11 class I conjugating enzymes and one class III enzyme. GL50803_5921 has a C-terminal extension of 170 amino acids and does not present any similarities within the protein database. One of the 12 genes classified in this group is a new finding; GL50803_8638 shares 43% identity with UBC6 from *Schizosaccharomyces pombe*.

The fourth group includes 82 sequences corresponding to E3 ligases. Five are HECT enzymes: GL50803_137754 and GL50803_17386 showed 53% and 54% similarity to human E6-AP, which is the founding member of the HECT family.^15^ GL50803_16321 is similar to Pub 1 from *S. pombe,* which ubiquitinates cdc25 (the mitotic phosphatase) *in vivo*.^15^ Furthermore, GL50803_32730 and GL50803_3117 are 31% identical to E3 ligases from *Caenorhabditis elegans* and *Eimeria maxima*, respectively (Table II).

**Fig. 3:**
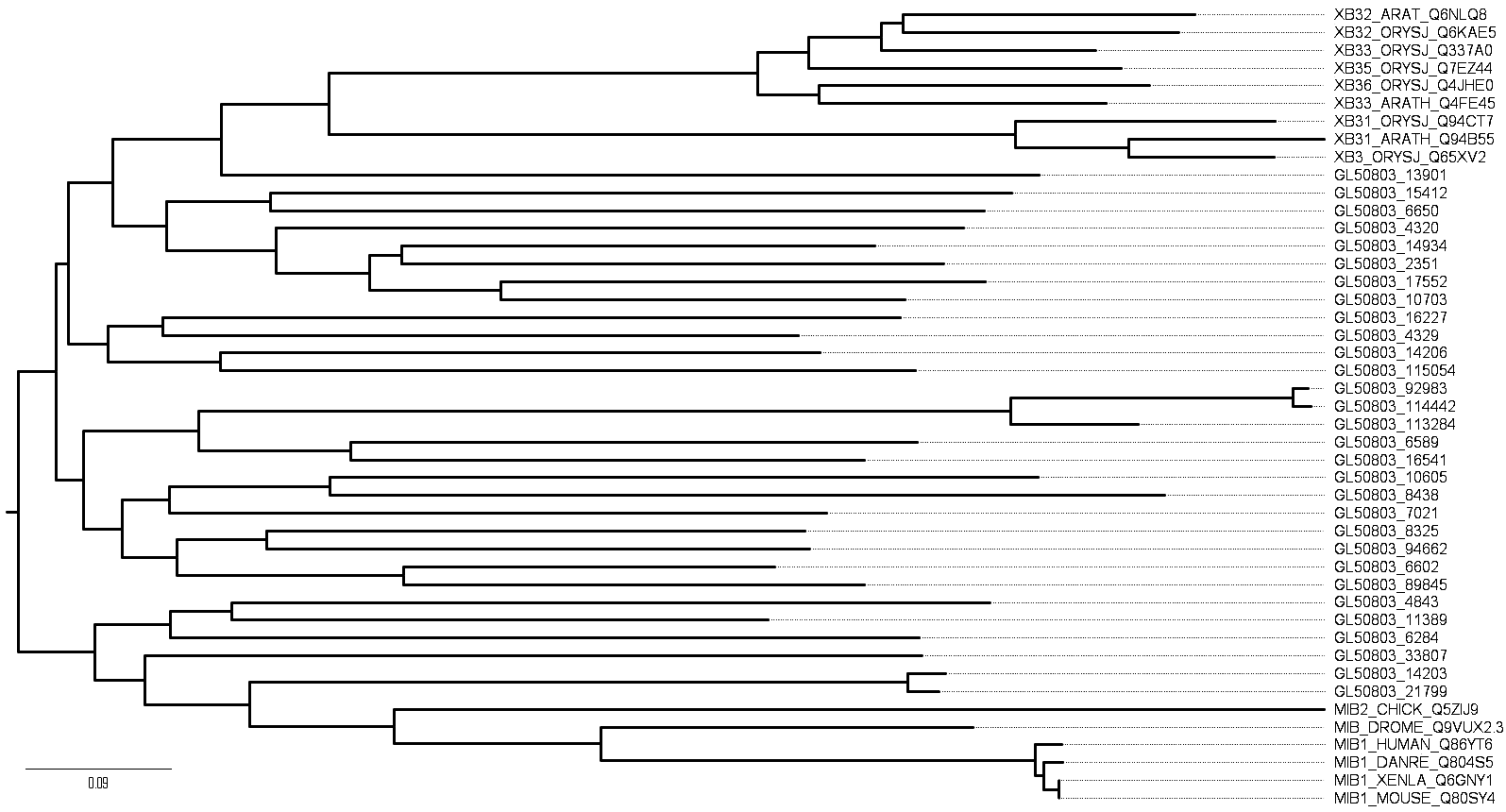
phylogenetic analysis of ankyrin-really interesting new gene (RING) E3 Ligases. Multiple sequence alignment was generated using CLUSTAL Omega; sequences from *Oryza sativa* (XB31,32,33,35,36), *Arabidopsis thaliana* (XB31,32,33), *Drosophila melanogaster* (MIB_DROME), *Mus musculus* (MIB1_MOUSE), *Xenopus laevis* (MIB1_XENLA), *Gallus gallus* (MIB2_CHICK), *Danio rerio* (MIB1_DANRE), and *Homo sapiens* (MIB1_HUMAN) were taken from UniProt. Accession numbers for the sequences are indicated. The phylogenetic tree was constructed using the MEGA7 program using the neighbor-joining method at 1000 bootstrap replicates.

The remaining sequences were classified as RING type, of which 71 displayed a typical C_3_HC_4_ domain. Within this class, we could distinguish three subgroups of metazoan E3 ligases. The first subgroup included 30 sequences containing ankyrin repeats and single C-terminal RING domain; this arrangement of domains has been reported in the XB3 family in plants.^16^ Although 14 of these proteins show some degree of similarity with Mind bomb (MIB) proteins, they do not conserve the typical modular architecture, including two substrate recognition modules at the N-terminal, a series of ankyrin repeats, and multiple RING domains at the C-terminal.^17^ Phylogenetic analysis was performed to determine whether *Giardia* ankyrin-RING proteins are members of the XB3 family. Our results revealed that the ankyrin-RING proteins were distributed into four distinct clusters, one of which appears to be related to XB3 (Fig. 3).

**Fig. 4:**
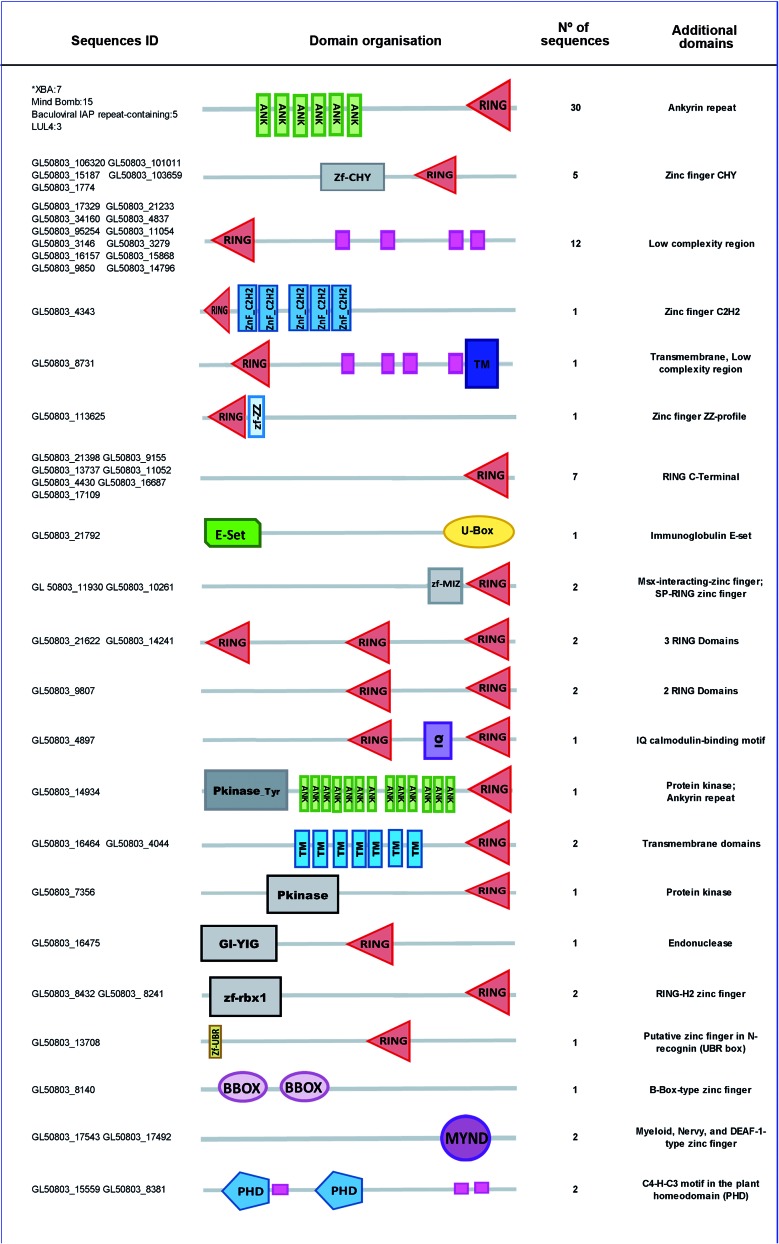
domain architecture of really interesting new gene (RING) E3 ligases. Domain architectures were predicted using the SMART database available at http://smart.embl-heidelberg.de/. The full protein is colored in gray; the low complexity region is indicated by the pink rectangle. The other domains identified are shown in different shapes and colours.

The second subgroup of RING ligases contains proteins with some degree of similarity to TRIM proteins, which are characterised by a tripartite motif composed of one RING domain, one or two B-Box domains, and a coiled-coil domain. TRIM proteins play important roles in various processes including cell growth, DNA damage signaling, senescence, tumor suppression, and innate antiviral response;^15^ the sequences identified share similarity with RING domains exclusively (Table II, Fig. 4).

In the last subgroup, there are several orthologs of ring finger and CHY zinc finger domain-containing protein 1 (RCHY1) also known as p53-induced protein with a RING-H2 domain (Pirh2); these proteins regulate cell-cycle progression, cell proliferation, and cell death through the ubiquitination and degradation of diverse substrates such as p53, p27Kip1, p63, p73, c-Myc, and Chk2.^15^

Furthermore, there were E3 ligases involved in nuclear functions, orthologs for Bre1, Pep5, and Hel 2 required for the degradation of histones in yeast. Orthologs for RAD18, Pirh2, and the endonuclease SLX1 involved in DNA damage repair. RNF12, E3 ligase for c-Myc, and orthologs to E3s, which engage in apoptosis and cellular signaling (e.g., IAP and XIAP), were also identified.

In contrast to the high number of sequences with a RING domain, only six harbor RING-related domains; one protein containing the Zf-UBR domain, which is involved in ubiquitination/degradation through the N-terminal rule; two proteins containing a B-Box domain; and two proteins with a PHD domain (C4HC3).

The fifth group corresponds to deubiquitinating enzymes; three new findings were reported: One deNEDDylase (GL50803_10218); an OTU enzyme (GL50803_88556), and a member of the MINDY-4 family (GL50803_7349). Other OTU enzymes have been reported in protozoans such as *Plasmodium falciparum, Cryptosporidium parvum*, *Toxoplasma gondii* and *Eimeria acervulina*,^18^ ^,^ ^19^ while orthologs of MINDY have not yet been reported in parasites.

**Fig. 5:**
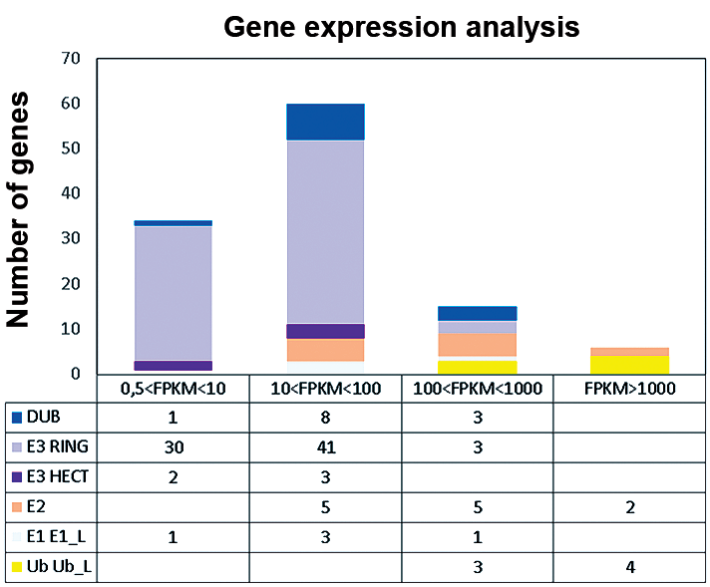
gene expression analysis. RNA-seq data of the trophozoite stage was evaluated. Genes with FPKM > 0.5 were classified into four groups according to their transcription levels.

Finally, to verify the expression of the genes identified during the trophozoite stage, transcriptomics data from Franzen et al.^20^ were used. These data were obtained from Illumina transcript analysis of WB strain assemblage A; genes with transcript levels formulated as fragments per kilobase per million fragments mapped (FPKM) < 0.5 were regarded as not expressed. From our analysis, transcription was detected in all of the genes but two (GL50803_16687 and GL50803_4430). FPKM ranged from 0.7 to 26.493, and a wide variation of expression levels within the five groups of the ubiquitination pathway genes was observed, with *ubiquitin*, *sumo*, *nedd8*, and *urm1* with the highest levels of transcripts (Fig. 5, Supplementary data). Although we analysed the data available from encystation,^21^ none of the genes were overexpressed during this differentiation process (data not shown).

## DISCUSSION

Approximately 30 genes associated with the Ub-conjugation pathway have been identified in *G. intestinalis*, 12 of which were previously identified at our laboratory using BLAST.^10^ This number is considerably lower than the hundreds of genes associated with this system reported in other eukaryotes. In humans, for example, there are two Ub activating enzymes (UBA1 and UBA6), approximately 40 conjugating proteins, and hundreds of E3 ligases.^1^ ^,^ ^3^ In this study, after an exhaustive search using the HMM, > 100 genes were identified.

In most organisms, Ub is codified in three forms: as a monomer, as polyUb, or as fusion proteins with ribosomal proteins. The Ub sequence is extensively conserved among all eukaryotes with similarities > 98% among humans, yeast, and apicomplexan parasites.^18^ However, *Giardia’s* Ub gene is one of the most divergent sequences reported; it shares only 70% similarity with the human sequence.^8^ ^,^ ^10^

Currently, the Ub-L family includes 10 members; some of them, such as FUB1, ISG15, and FAT10, are exclusive to metazoan and are involved in the immune response, T cell activation, and antiviral and antimicrobial defense, respectively.^2^ Here, we identified SUMO, NEDD8, URM1, and UFM1 homologs; however, we did not identify ATG8 and HUB1. Orthologs of HUB1 have been identified from yeasts to humans, in apicomplexan parasites, and in *Entamoeba,* (deep-branching eukaryote, such as *Giardia*).^11^ ^,^ ^18^ ^,^ ^22^ In *S. cerevisiae*, *S. pombe*, and humans, HUB1 is mainly involved in alternative splicing of pre-mRNA.^23^ Notably, in *Giardia*, few introns have been reported, and splicing is not essential for the parasite’s viability.^24^

ATG8 plays a central role in the autophagy network, and its conjugation requires the activities of the ATG7 (activating) and ATG3 (conjugating) enzymes. In related organisms such as *Entamoeba*, *Cryptosporidium*, and *Trichomonas vaginalis* and in other protozoa parasites*,* the ATG8 system has been identified, and autophagy plays a principal role in the parasites’ survival.^11^ ^,^ ^18^ ^,^ ^22^ In this study, ATG8, ATG7, and ATG3 enzymes were not identified, as previously reported by Bagchi et al.,^25^ who suggested that in *Giardia,* autophagy does not operate by the classical mechanism based on these proteins.

UFM1 is a highly conserved protein in metazoa and plants but not in yeasts. Initially, this modification was considered to be metazoa-specific and was associated with the endoplasmic reticulum stress response; however, recently, Gannavaram et al.^26^ demonstrated the existence of UFM1 and its conjugation enzymes: UBA5 (E1), UFC1 (E2), and UFL1 (E3) in *Trypanosomatidae* parasite proteomes. In *Leishmania donovani*, is associated with the mitochondria, and plays an important role in pathogenesis.^26^ UFM1 homologs have not been identified in other unicellular parasites; although we identified a UFM1 domain-containing protein (GL50803_104982) with a high E-value (2.27e-30), we did not find the conjugation enzymes and the predicted structure was not typical for Ub-L (Fig. 1).

URM1 acts as a protein modifier (urmylation) and belongs to the superfamily MoaD and ThiS in prokaryotes; these are small sulfur carrier proteins involved in molybdenum cofactor (MoaD) and thiamin (ThiS) biosynthesis, respectively. The URM1 sequence and structure are similar to those of bacterial proteins than those of Ub-Ls, and its conjugation process depends entirely on UBA4.^14^ We identified URM1, UBA4, and bacterial MoeB homologs; however, we did not identify MoaD or ThiS protein (Table II). The sequences for UBA4 and MoeB share approximately 24% sequence identity, and they are differentially expressed during encystation and temperature or redox stress response (data available at www.giardiadb.org), suggesting that they are involved in two different processes. However, whether URM1 can be activated by two enzymes, or if there is another member of the MoaD/ThiS family to be identified, is yet unclear.

Among Ub-Ls, NEDD8 has the highest identity with Ub (approximately 60%) and is highly conserved from yeast to humans. NEDDylation is catalysed by the specific enzymes: UBA3, UBE2F, UBC12, RING-box proteins (Rbx1 and Rbx2) and is reversed by specific proteases, such as DEN1/SENP8 and UCH L3.^2^ This conjugation cascade has been identified in other protozoans, such as *Plasmodium spp*, *T. gondii*, *C. parvum*, *Entamoeba spp*, and *Trypanosoma brucei*.^18^ ^,^ ^27^ Although cullins are the most abundant substrates, proteins such as DNA damage binding protein 1 (DDB1), translation elongation factor α1, the chaperone DnaJ, in addition to the NEDDylation enzymes have been reported as targets.^27^ In this study we found the NEDD8 ortholog and enzymes involved in the conjugation and de-NEDDylation processes (Table II).

The covalent modification of proteins by SUMO is the unique ubiquitination-like process that was described previously in *Giardia*; a single gene for SUMO, SUMO activating Enzyme subunit 2 (SAE2), SUMO conjugating enzyme (Ubc9), and one deSUMOylase were reported by Vranych et al.^13^ The same sequences were identified here; however, this finding differs from that of higher eukaryotes, in which multiple members of the SUMO family exist and SAE2 is a heterodimer of UBA1 and AOS1 subunits.^2^ Although only two SUMOylated proteins, arginine deiminase and α-tubulin have been fully identified in *Giardia*, SUMOylation of other proteins and the participation of SUMOylation in encystation, cell-cycle progression, cell growth, and morphology maintenance were recently demonstrated.^28^

Regarding the ubiquitination process, the extensive conservation exhibited by the E2 enzymes enabled the identification of most of *Giardia*’s enzymes using basic local alignment tools in the past;^10^ however, a novel noncanonical ubiquitin-conjugating enzyme (NCUBE) was identified here (GL50803_8638); NCUBE enzymes are localised in the lumen of endoplasmic reticulum (ER) and participate in ER-associated degradation. Nevertheless, *Giardia*’s protein and one ortholog of *Entamoeba* lack the hydrophobic C-terminal tail required for ER localisation; the function of this “truncated” Ubc6-like protein has not yet been established.^29^

The E3 Ub ligase family is the largest family of proteins involved in ubiquitination because it is required for specific substrate recognition. In *Giardia*, we identified approximately 80 enzymes, with five containing the HECT domain; our results agree with those reported for *Entamoeba*, apicomplexan, and yeast, which are organisms that codify for five or six HECT ligases.^22^

Considering the RING E3 ligases, our results largely differ from those previously reported by Gallego et al.;^10^ they identified only one putative protein with the RING domain, whereas we identified approximately 70. Similar to other eukaryotes, in *Giardia*, ubiquitination is involved in numerous cellular processes such as protein quality control, metabolic pathways, endocytosis, cell signaling, DNA/RNA metabolism, and differentiation.^6^ The large and diverse group of RING ligases reported here corresponds to multiple processes regulated by ubiquitination.

The over-representation of members of the XB3 family (Ankyrin repeat C_3_HC_4_ RING finger) (Figs 3, 4) suggests an important role in parasite growth; this type of protein has not been reported in other parasites or in yeast. Our data provide the first reference for the XB3 family in a single-cell eukaryote. In plants, these proteins play important roles in development, stress responses, cell death induction, and pathogen response.^16^

Another interesting result is the absence of multisubunit E3 enzymes, which are important cell-cycle regulators in higher eukaryotes.^30^ The anaphase promoter complex (APC) and SCF are E3 ligases that ubiquitinate cyclins involved in the M and G1/S phases, respectively. The APC/C holoenzyme comprises at least 14 different proteins distributed into three subcomplexes: a scaffolding subcomplex, a catalytic subcomplex (containing APC11 RING ligase, cullin-like subunit APC2, and APC10), and the substrate recognition subcomplex.^30^ Our search identified only one small protein (GL50803_8432; 78 amino acids) that is 44% similar to the APC11 from *Mus musculus* (84 amino acids). The SCF complex consists of three subunits: Skp1/Cullin1 (scaffolding protein), Rbx1 (RING Ligase), and an interchangeable F-box-protein that determines substrate specificity. This E3 complex ubiquitinates the S phase CDK inhibitor (Sic1p/p27kip1) involved in G1/S phase progression.^30^ Our search failed to identify any sequence for Skp1 and Cullin1 homologs, although two Rbx proteins were identified (GL50803_8432 and GL50803_8241). These results agree with those reported by Eme et al.^31^ and Gourguechon et al.;^32^ none of them found any of the highly conserved components of these Ub ligases in *Giardia*. In addition, Gourguechon’s study showed that APC substrates as mitotic cyclin B, aurora and polo-like kinases were not ubiquitinated and that proteasome inhibition did not cause cell-cycle arrest, suggesting that no ubiquitination and protein degradation via proteasome are involved in *Giardia* cycle progression.

In 1994, Krebber suggested that the ubiquitination system in *Giardia* was a basal acquired system;^8^ nonetheless, the set of genes reported here demonstrates that the *Giardia* genome codifies members of the Ub and Ub-L conjugation system, similar to that described for higher eukaryotes.
