# Supplementary material for: A new gene inventory of the ubiquitin and ubiquitin-like conjugation pathways in Giardia intestinalis
Source: Mem Inst Oswaldo Cruz. 2020 Feb 21;115:e190242. doi: 10.1590/0074-02760190242 (PMC7029713; doi:10.1590/0074-02760190242)
Supplement: Supplementary file 1 [file 1678-8060-mioc-115-e190242-s.pdf]

Gene Expression Analysis

	ID	0,5<FPKM<10
UBA2	GL50803_6288	3,6
E3 HECT	GL50803_17386	4,5
E3 HECT	GL50803_3117	3,9
E3 RING	GL50803_16687	0,0
E3 RING	GL50803_4430	0,1
E3 RING	GL50803_15559	0,7
E3 RING	GL50803_101011	0,8
E3 RING	GL50803_106320	0,9
E3 RING	GL50803_14203	4,5
E3 RING	GL50803_4320	2,9
E3 RING	GL50803_10605	7,2
E3 RING	GL50803_6602	6,6
E3 RING	GL50803_89845	3,7
E3 RING	GL50803_115054	4,7
E3 RING	GL50803_13901	3,7
E3 RING	GL50803_8325	4,6
E3 RING	GL50803_14206	7,7
E3 RING	GL50803_94662	3,6
E3 RING	GL50803_6589	3,9
E3 RING	GL50803_114442	3,7
E3 RING	GL50803_4837	1,5
E3 RING	GL50803_15187	8,0
E3 RING	GL50803_103659	1,4
E3 RING	GL50803_15868	7,7
E3 RING	GL50803_9850	3,6
E3 RING	GL50803_9155	6,0
E3 RING	GL50803_13737	7,7
E3 RING	GL50803_11052	2,2
E3 RING	GL50803_21792	7,4
E3 RING	GL50803_11930	3,0
E3 RING	GL50803_10261	2,2
E3 RING	GL50803_21622	9,9
E3 RING	GL50803_4897	3,3
E3 RING	GL50803_16464	6,2
E3 RING	GL50803_16475	6,1
E3 RING	GL50803_13708	2,7
DUB	GL50803_16438	5,5

	ID	10<FPKM<100
E1	GL50803_10661	32,2
UBA3	GL50803_4083	39,0
UBA4	GL50803_12853	58,9
E2	GL50803_15162	51,5
E2	GL50803_6524	50,8
E2	GL50803_5921	78,4
E2	GL50803_31576	10,2
E2	GL50803_8638	87,1
E3 HECT	GL50803_137754	55,9
E3 HECT	GL50803_16321	21,6
E3 HECT	GL50803_32730	42,3
E3 RING	GL50803_16541	22,6
E3 RING	GL50803_4843	50,5
E3 RING	GL50803_16227	77,6
E3 RING	GL50803_17552	20,1
E3 RING	GL50803_2351	10,1
E3 RING	GL50803_6284	48,5
E3 RING	GL50803_21799	15,8
E3 RING	GL50803_33807	15,1
E3 RING	GL50803_4329	15,4
E3 RING	GL50803_8438	85,0
E3 RING	GL50803_15412	11,9
E3 RING	GL50803_6650	14,3
E3 RING	GL50803_11389	16,8
E3 RING	GL50803_7021	78,7
E3 RING	GL50803_92983	21,1
E3 RING	GL50803_17329	45,4
E3 RING	GL50803_21233	51,8
E3 RING	GL50803_34160	75,8
E3 RING	GL50803_95254	11,6
E3 RING	GL50803_1774	26,5
E3 RING	GL50803_11054	22,4
E3 RING	GL50803_3146	17,0
E3 RING	GL50803_3279	10,1
E3 RING	GL50803_16157	58,2
E3 RING	GL50803_14796	44,4
E3 RING	GL50803_4343	15,7
E3 RING	GL50803_95918	10,4
E3 RING	GL50803_8731	20,1
E3 RING	GL50803_113625	12,2
E3 RING	GL50803_21398	29,0
E3 RING	GL50803_17109	39,4
E3 RING	GL50803_9807	12,5
E3 RING	GL50803_14241	28,9
E3 RING	GL50803_14934	13,3
E3 RING	GL50803_4044	40,8
E3 RING	GL50803_7356	17,8
E3 RING	GL50803_8432	20,1
E3 RING	GL50803_8241	55,9
E3 RING	GL50803_8140	59,1
E3 RING	GL50803_17492	20,7
E3 RING	GL50803_8381	28,0
DUB	GL50803_16090	43,0
DUB	GL50803_5533	98,2
DUB	GL50803_6317	20,4
DUB	GL50803_102710	37,9
DUB	GL50803_10218	11,6
DUB	GL50803_16823	97,4
DUB	GL50803_24425	41,0
DUB	GL50803_7349	15,6

	ID	100<FPKM<1000
Ub	GL50803_8843	188,5
URM	GL50803_11884	941,7
UFM	GL50803_104982	100,6
MOEB	GL50803_11436	598,0
E2	GL50803_3978	438,1
E2	GL50803_27055	172,4
E2	GL50803_3171	188,4
E2	GL50803_2876	341,0
E2	GL50803_24068	156,7
E3 RING	GL50803_10703	114,3
E3 RING	GL50803_113284	340,8
E3 RING	GL50803_17543	152,0
DUB	GL50803_14460	174,5
DUB	GL50803_8189	102,0
DUB	GL50803_88556	152,8

		FPKM>1000
UbL40	GL50803_5665	2.690,6
UbS27	GL50803_16298	19.213,2
SUMO	GL50803_7760	2.392,3
NEDD8	GL50803_7110	26.493,8
E2	GL50803_15252	2.590,7
E2	GL50803_12950	1.695,8

CC BY
